# The Most Common *VHL* Point Mutation R167Q in Hereditary VHL Disease Interferes with Cell Plasticity Regulation

**DOI:** 10.3390/cancers13153897

**Published:** 2021-08-02

**Authors:** Stéphanie Buart, Stéphane Terry, M’boyba Khadija Diop, Philippe Dessen, Sophie Couvé, Abdérémane Abdou, Julien Adam, Jérôme Thiery, Pierre Savagner, Salem Chouaib

**Affiliations:** 1 INSERM UMR 1186, Integrative Tumor Immunology and Immunotherapy, Gustave Roussy, Faculty of Medicine, University Paris-Saclay, 94805 Villejuif, France; stephanie.buart@gustaveroussy.fr (S.B.); stephane.terry@gustaveroussy.fr (S.T.); abderamane.abdou@gustaveroussy.fr (A.A.); jadam@ghpsj.fr (J.A.); jerome.thiery@gustaveroussy.fr (J.T.); pierre.savagner@gustaveroussy.fr (P.S.); 2 Bioinformatics Core Facility, University of Paris-Saclay, 94805 Villejuif, France; Mboyba.diop@gustaveroussy.fr (M.K.D.); philippe.dessen@gustaveroussy.fr (P.D.); 3 EPHE, PSL Université, 75006 Paris, France; sophie.couve@gustaveroussy.fr; 4 CNRS UMR 9019, Gustave Roussy, University Paris-Saclay, 94805 Villejuif, France; 5 Biology and Pathology Department, University Paris-Saclay, 94805 Villejuif, France; 6 Thumbay Research Institute for Precision Medicine, Gulf Medical University, Ajman 4184, United Arab Emirates

**Keywords:** ccRCCs, R167Q VHL mutation, hypoxia-related genes, stemness-related genes, ccRCC patients survival

## Abstract

**Simple Summary:**

Von Hippel–Lindau (*VHL*) disease is characterized by mutations in the *VHL* gene, which can induce numerous benign and malignant tumors in different organs, as well as highly vascularized clear cell renal cell carcinomas (ccRCCs). The aim of this study was to examine whether the VHL-R167Q mutation, which is associated with a high risk of developing ccRCC (type 2B VHL disease), could impact the plasticity of renal carcinoma cells. Our transcriptomic results show that VHL-R167Q regulates hypoxia-, plasticity-, and stemness-related genes. Moreover, analysis in a ccRCCs TCGA dataset highlighted that a number of genes regulated by hypoxia and related to stemness in VHL-R167Q-expressing tumors are associated with a poor survival in ccRCCs patients.

**Abstract:**

Von Hippel–Lindau *disease* (*VHL*) is a rare hereditary syndrome due to mutations of the *VHL* tumor suppressor gene. Patients harboring the R167Q mutation of the *VHL* gene have a high risk of developing ccRCCs. We asked whether the R167Q mutation with critical aspects of pseudo-hypoxia interferes with tumor plasticity. For this purpose, we used wild-type *VHL* (WT-VHL) and VHL-R167Q reconstituted cells. We showed that WT-VHL and VHL-R167Q expression had a similar effect on cell morphology and colony formation. However, cells transfected with VHL-R167Q display an intermediate, HIF2-dependent, epithelial–mesenchymal phenotype. Using RNA sequencing, we showed that this mutation upregulates the expression of genes involved in the hypoxia pathway, indicating that such mutation is conferring an enhanced pseudo-hypoxic state. Importantly, this hypoxic state correlates with the induction of genes belonging to epithelial–mesenchymal transition (EMT) and stemness pathways, as revealed by GSEA TCGA analysis. Moreover, among these deregulated genes, we identified nine genes specifically associated with a poor patient survival in the TCGA KIRC dataset. Together, these observations support the hypothesis that a discrete VHL point mutation interferes with tumor plasticity and may impact cell behavior by exacerbating phenotypic switching. A better understanding of the role of this mutation might guide the search for more effective treatments to combat ccRCCs.

## 1. Introduction

Von Hippel–Lindau (*VHL*) disease is a rare hereditary cancer syndrome caused by germinal mutations of the *VHL* tumor suppressor gene. VHL disease is classified in two types (type 1 and type 2), which are based on the presence of pheochromocytoma. Type 1 VHL patients have a low risk of developing pheochromocytoma, while type 2 VHL patients have high risk for developing pheochromocytoma. Furthermore, the type 2 subset is subdivided into type 2A, 2B, and 2C subtypes based on the risk of developing clear cell renal cell carcinomas (ccRCCs) in addition to pheochromocytoma. Patients with type 2A have low risk, type 2B have high risk, and 2C have no risk of developing this malignancy [1].

Clear cell RCC, the most prevalent RCC type, is refractory to chemotherapy and radiation therapy [2]. It is frequently associated with alterations of the tumor suppressor VHL gene [3]. In these tumors, *VHL* can be altered by germline or somatic mutations, together with a loss of heterozygosity (LOH) or promoter hypermethylation [4], which leads to quantitative or qualitative alterations of the VHL protein (pVHL) functions. One of the most important roles of pVHL has been well established as the targeted binding of E3 ubiquitin ligase to the α subunit of hypoxia inducible factors (HIF-α) when prolyl residues 402 and 564 are hydroxylated for oxygen-dependent proteolysis [5,6,7]. In normoxia, cells that are deficient in pVHL contain high levels of HIF-α. HIF-α is the main regulator of the transcriptional activation of hypoxia-inducible genes, which are implicated in renal carcinogenesis. In this regard, W.G. Kaelin, P.J. Ratcliffe, and G.L. Semenza have discovered how cells sense and adapt to oxygen availability [8]. Thus, *VHL* alterations in 70–90% of sporadic ccRCCs result in the dysregulation of HIF downstream genes [9]. Consequently, these alterations play a crucial role in the modification of signaling networks and tumor pathogenesis, and RCC patients with the *VHL* mutation are characterized by having a poor prognosis [10].

As mentioned above, a mutated or lost *VHL* gene prevents pVHL from participating in E3 ubiquitin ligase complex formation and interaction with its HIF-α substrate, allowing stabilization of HIF-α even in physiological levels of oxygen [11]. This phenomenon is called “pseudo-hypoxia” and promotes cellular mechanisms following VHL mutation that resemble those induced during exposure to non-physiological low pO_2_ levels. Indeed, similar to hypoxia, pseudo-hypoxia, exacerbates cancer progression and maintains a deep control on the epithelial–mesenchymal-transition (EMT), characterized by a strong immunosuppression [12] and a strong disturbance of microenvironmental conditions. Indeed, it is widely appreciated that the majority of malignancies create a hostile hypoxic microenvironment that can hamper cell-mediated immunity and dampen the efficacy of the immune response. Among the microenvironmental factors that play a dominant role in neoplasia, hypoxia is thus believed to be one of the most relevant in the neoplastic response of tumor cells [13]. It is an integral component of the tumor microenvironment and especially of the pathologically vascularized zones inside solid tumors, contributing to immune tolerance of tumor cells by impeding the homing of immunocompetent cells into tumors and inhibiting their antitumor efficacy [14]. In this regard, the VHL protein has been associated with several cellular activities, including hypoxia response, cell cycle arrest, autophagy, apoptosis, and extracellular matrix remodeling. In ccRCCs, evidence indicates that the loss of VHL function leads to the constitutive stabilization of hypoxia-inducible factors (HIFs), in particular HIF-2α, resulting in a highly angiogenic environment in these extremely vascularized and chemo-radio-resistant tumors [15]. Approximately half of VHL patients have VHL missense mutations, and type 2 VHL disease is mainly characterized by missense mutations, with codon 167 being considered as the mutation hot spot [16]. The R167Q mutation (VHL-R167Q) disrupts VHL binding with elongin C and therefore disrupts the functional VHL-elongin B-elongin C (VBC) E3 ligase complex [17,18]. Although there have been studies investigating this mutation, the manner by which the mutation contributes to tumorigenesis is not fully understood.

In this work, our aim was to get a better understanding of the impact of the VHL-R167Q mutation on RCC transcriptional regulation and plasticity. We demonstrated that the protein level of VHL-R167Q, which has the functional capacity to downregulate HIF-2α, interferes with the transcription of genes belonging to cancer cell stemness and tumor plasticity pathways in 786-0 R167Q cells, as compared with 786-0 EV cells.

## 2. Materials and Methods

### 2.1. Mutated VHL Cell Lines

The 786-0 cell line was isolated from a VHL-/- human ccRCC [19]. The three different VHL mutated cell lines used in this study were generated in the laboratory of S. Richard (Gustave Roussy, Villejuif, France) and were already described [18]. Briefly, 786-0 cells were transfected with a vector allowing the doxycycline-inducible expression of the full-length wild-type sequence of *VHL* (786-0 VHL WT, VHL+/+) or the sequence encoding the R167Q mutant (786-0 R167Q, VHL mutated) [18]. Control cells were generated by the transfection of the empty vector (786-0 EV, VHL-/-).

The three cell lines were maintained in culture in DMEM medium (Gibco, Thermo Fisher Scientific, Inc., Waltham, MA, USA), supplemented with 5% tetracycline-free fetal calf serum (FCS) (PAA Laboratories Inc, Ontario, Canada), 1% sodium pyruvate, 1% penicillin/streptomycin, 0.2 mg/mL zeocin, 3 μg/mL blasticidin, and 1 μg/mL doxycycline for two weeks to allow VHL expression. These cells lines were genotyped by direct sequencing of *VHL* after reception in our laboratory.

### 2.2. Western Blots

Cell lines were washed twice in phosphate-buffered saline and lysed with RIPA buffer (Sigma-Aldrich, St. Louis, MO, USA) supplemented with a protease and phosphatase inhibitor cocktail (Thermo Fisher Scientific Inc., Waltham, MA, USA). Lysates were sonicated on ice, resolved by sodium dodecyl sulfate-polyacrylamide gel electrophoresis, and transferred onto nitrocellulose membranes. The membranes were blocked in blocking buffer, then they were probed overnight at 4 °C with the following primary Abs: anti-VHL (Mouse Ab, 556347, BD Biosciences, Franklin Lakes, NJ, USA), HIF-2α (Rabbit Ab, 7096S, Cell Signaling Technology; Danvers, MA, USA), E-cadherin (Rabbit Ab, 3195S, Cell Signaling Technology; Danvers, MA, USA), vimentin (Mouse Ab, SC6260, Santa Cruz Biotechnology, Dallas, TX, USA), ZEB1 (Mouse Ab, SC51797, Santa Cruz Biotechnology, Dallas, TX, USA), AXL total (Rabbit Ab, 8661S, Cell Signaling Technology, Danvers, MA, USA), and β-actin (Mouse Ab, clone AC-15, Sigma-Aldrich, St. Louis, MO, USA). The labeling was performed following incubation with horseradish peroxidase (HRP)-conjugated secondary Abs and detection with an enhanced chemiluminescence kit (GE Healthcare, Chicago, IL, USA). Some blots were scanned and some blot images were captured using a ChemiDoc Imaging System (Biorad, Hercules, CA, USA). Western blot quantifications were performed using ImageJ densitometry software (NIH, Bethesda, MD, USA). 

### 2.3. Phase Contrast and Fluorescence Microscopy

A total of 40,000 cells were grown on glass coverslips (Sigma-Aldrich, St. Louis, MO, USA) and fixed for 20 min in PBS/2% PFA, washed, and incubated 20 min in PBS/50 mM NH4Cl. Cells were washed with PBS, and surface area was estimated using the “measurement” tool from ImageJ software. More than 50 cells from at least three different optical fields were examined for each cell line. For co-staining HIF2α and VHL, multiplexed fluorescent IHC was performed on 4 μm FFPE cell line sections using Ventana Benchmark and Discovery automated platforms by sequential staining with a monoclonal rabbit anti-HIF2 α (7096S, 1:25, Cell Signaling Technology, Danvers, MA, USA) and then a monoclonal mouse anti-VHL (556347, 1:100, BD Biosciences, Franklin Lakes, NJ, USA). Briefly, after deparaffinization and epitope retrieval in CC1 buffer (pH = 7,8, 32 min at 95 °C), tissue sections were incubated with the first primary Ab for anti-HIF-2 α for 1 h at room temperature, then incubated with an HRP-conjugated amplification system associated with a tyramide-coupled fluorophore: Opal 520 (1:200). Tissue sections were then incubated with the second primary Ab for anti-VHL for 1h at room temperature, then incubated with a tyramide-coupled fluorophore: Opal 690 (1:200). Multispectral fluorescent images were captured using the Olympus scanner VS200 and analyzed with OlyVIA software. For immunofluorescence vimentin and ZEB1, cells were permeabilized for 5 min in PBS/0.2% Triton X-100. After 2 washes in PBS, coverslips were placed in blocking solution (PBS/10% FCS) for 30 min, washed once in PBS and incubated for 1 h at RT with the Ab of interest diluted in incubation buffer (PBS/0.05% Triton X-100). The different Abs used were a mouse monoclonal anti-vimentin (Clone V9, SC6260, Santa Cruz Biotechnology, Dallas, TX, USA) and a mouse monoclonal anti-ZEB1 (SC51797, Santa Cruz Biotechnology, Dallas, TX, USA). Cells were then washed 3 times with incubation buffer and incubated 1 h at RT with goat Alexa-Fluor 488 or Alexa-Fluor 555-conjugated secondary antibody (Life Technologies Thermo Fisher Scientific, Inc., Waltham, MA, USA) in incubation buffer containing 5% FCS. Cells were then washed 3 times in PBS and mounted in Vectashield mounting medium (Vector Laboratories, Eurobio Scientific, Les Ulis, France) before imaging (IX83 microscope, Olympus, Shinjuku, Tokyo, Japan) and analysis (CellSense Dimension software, Olympus, Shinjuku, Tokyo, Japan).

### 2.4. Clonogenic Assay

Cells were detached then counted. A total of 500 viable cells were plated on 6-well cell culture plates. After 9 days of culture, cells were washed in PBS, then fixed and stained during 30 min with a solution containing 25% methanol and 0.01% crystal violet. Crystal violet binds to DNA in the nuclei of mammalian cells, staining them a deep purple and helping to visualize colonies. Upon removal of the fix/stain solution, cells were rinsed carefully with distilled water. The colonies were allowed to dry at room temperature. Photographs of the 6-well cell plates for each cell line were taken with a ChemiDoc imaging system (Biorad, Hercules, CA, USA).

### 2.5. Immunohistochemistry Staining for AXL

Cells were detached, then washed twice in PBS. Cell lines were fixed in 4% PFA, then included in paraffin. Sections of four micrometer of fixed-formaldehyde paraffin embedded (FFPE) cell lines were deparaffinized and rehydrated. Sections were treated with antigen retrieval solution (citrate buffer, pH 7.3, concentrated 10×, T0130, Diapath, Martinengo, Italy) in water bath at 95 °C for 30 min. Tissue sections were then incubated for 10 min with 3% H_2_O_2_ and PowerVision IHC/ISH Super Blocking PV6122 solution (MM France, Brignais, France) for 10 min. Histological slides were incubated overnight at 4 °C with a rabbit anti-human AXL total mAb (8661S, Cell Signaling Technology; Danvers, MA, USA) or a rabbit isotype control Ab (3900, Cell Signaling Technology; Danvers, MA, USA). For signal amplification, slides were then incubated with a rabbit primary antibody (Polink-2 plus HRP detection, 39–18, Diagomics, Blagnac, France). The signal was revealed with DAB chromogene and Mayer’s hemalum solution counterstain (Merck Millipore, Billerica, MA, USA). This analysis was considered as a positive membrane and/or cytoplasmic immunostaining of the total AXL in cancer cells.

### 2.6. Flow Cytometry Analysis

Phenotypic analyses of the three cell lines were performed by direct immuno-staining. Briefly, 0.2 × 10^6^ cells were stained with the following Abs: APC-conjugated mouse anti-AXL monoclonal (Clone 108724, R&D Systems, Minneapolis, MN, USA), PE-conjugated mouse anti-human PD-L1 (Clone 29E.2A3, Biolegend San Diego, CA, USA); APC-conjugated mouse anti-human N-cadherin (Clone 8C11, Biolegend San Diego, CA, USA); PE-conjugated mouse anti-human E-cadherin (67A4, Biolegend San Diego, CA, USA). Extracellular staining was performed at 4 °C. Acquisitions were performed using a BD Accuri™ C6 flow cytometer (BD Biosciences, Franklin Lakes, NJ, USA), and data were processed using the FlowJo program (Becton, Dickinson, gAshland, OR, USA).

### 2.7. RNA-Sequencing and Gene Set Enrichment Analysis

Total RNA extraction for the gene set enrichment analysis (GSEA) study was performed with an RNA/DNA purification kit (Norgen, UK). RNA sequencing and analysis were performed as described in [20]. Briefly, RNA integrity (RNA integrity score >8) was checked on the Agilent 2100 Bioanalyzer. An amount of 50 ng of total RNA was processed for poly-A mRNA selection using oligo(dT) beads and subjected to mRNA fragmentation. Subsequently, dsDNA libraries were generated from the fragmented mRNAs, bar-coded, purified, and subjected to paired-end sequencing on a HiSeq-2000 sequencer (Illumina, San Diego, CA, USA). For quality control assessment, FastQC (v 0.11.3) was used. Read counting over the transcriptome was carried out using Salmon (v 0.8.2) [21] and Gencode (v 19, GRCh37.p13) with default parameters. Differential analysis was performed using wasabi (v 0.1), sleuth (v 0.28.1) [22], and in-house scripts within an R (v 3.2.3) environment. Clustering, PCA, and volcano plot analyses were conducted to explore gene expression variations. GSEA was performed with the GSEA platform of the Broad Institute, following guidelines (http://www.broadinstitute.org/gsea/index.jsp (accessed on 18 November 2019).

### 2.8. Analysis of Gene-Expression Data Derived from The Cancer Genome Atlas

RNA-Seq expression data and sample information on renal clear cell carcinoma from The Cancer Genome Atlas (KIRC-TCGA) were accessed in June 2020 from cBioPortal [23]. Cases with available expression data and follow-up information (*n* = 500) were considered. To explore the prognostic value of the genes, cases were stratified into two groups (low and high expression) based on the expression of individual genes and using the median cutoff. Kaplan–Meier plots were then generated using GraphPad Prism versions 8 to integrate overall survival information.

## 3. Results

### 3.1. VHL-R167Q and WT VHL Display Similar Slight Effect on Cell Morphology and Colony Formation, Whereas VHL-R167Q Promotes HIF-2 Expression

**Figure 1:**
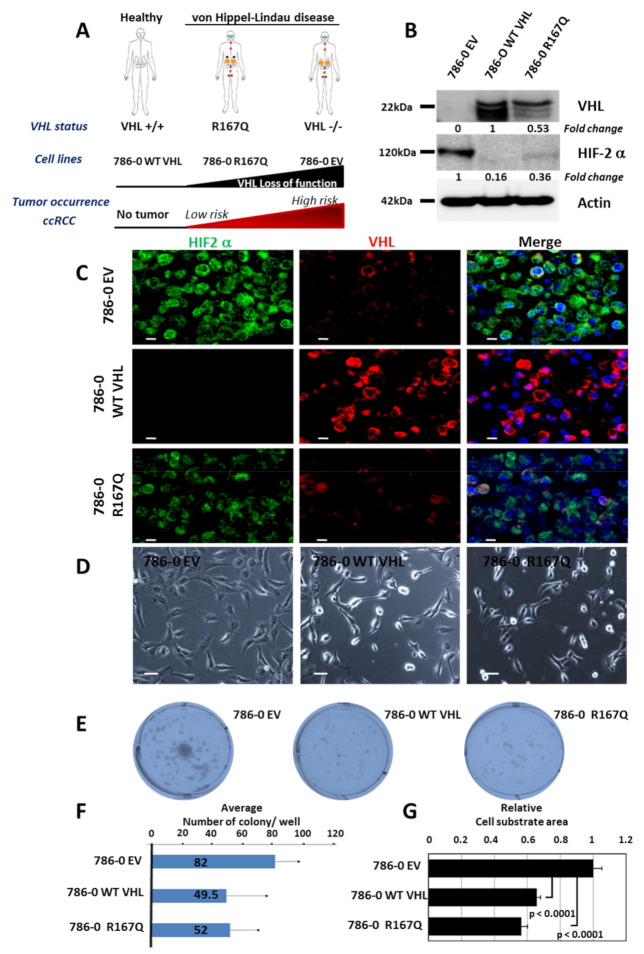
Similar cell morphology and colony formation for WT VHL and VHL-R167Q, whereas VHL-R167Q slightly promotes HIF-2α. (**A**): Schematic representation of the 786-0 model and the consequences of VHL alterations on tumor occurrence (adapted from S. Couvé et al. [18]). VHL disease predisposes to ccRCC and pheochromocytomas (pheo) in patients, depending on the loss of function of VHL, with an ascending correlation. VHL disease is characterized by the development of tumors (circles): CNS hemangioblastomas that can occur anywhere along the brain–spine areas (red), retinal hemangioblastomas (blue), pheochromocytomas (black) and ccRCC (yellow) with a low (small circle) or high risk (large circle), pancreatic cysts and neuroendocrine tumors (purple), and epididymal cystadenomas (brown). Three cell lines were derived from the ccRCC 786-0 VHL-/- cell line transfected with a vector doxycycline inducible with the wild-type sequence of VHL (786-0 WT VHL) or the mutated sequence VHL R167Q (786-0 VHL-R167Q). Each type of phenotype is indicated and the corresponding loss of function of the mutants is represented by a black triangle. Tumor occurrence is indicated below by a red triangle. (**B**): Western blot analysis of VHL and HIF-2α proteins from 786-0 EV, 786-0 WT VHL, and 786-0 R167Q. Actin is used as a loading control, the uncropped western blot figure in Figure S2. (**C**): Immunofluorescence staining of HIF-2α and VHL in 786-0 EV, 786-0 WT VHL, and 786-0 R167Q cells. Scale bar, 10 μm. (**D**): Phase contrast microscopy images showing the similar morphology of the three cells lines, 786-0 EV, 786-0 WT VHL, and 786-0 VHL-R167Q. Scale bar, 50 μm. (**E**): Clonogenic assay using 786-0 EV, 786-0 WT VHL, and 786-0 VHL-R167Q cell lines. The photograph corresponds to one representative well. (**F**): The count of the colony is the mean ± SD of nine wells from two independent experiments for each cell lines. (**G**): Surface area covered by 786-0 EV, 786-0 WT VHL, and 786-0 VHL-R167Q cells. More than 50 cells from each cell line were examined in at least three different fields, using ImageJ software to evaluate cell spreading. *p* values were determined with Student’s *t* tests.

It is well established that mutations of *VHL* lead to morphologically different RCCs with distinct clinical courses and outcomes. It is also admitted that a loss of *VHL* results in a pseudo-hypoxic state and the subsequent activation of cellular response pathways mediated by HIF-2, despite normal oxygen conditions. In a previous study, Couvé et al. demonstrated that the complex pattern of disease manifestations observed in VHL syndrome is perfectly correlated with a gradient of pVHL dysfunction in hypoxia signaling pathways [18]. We took advantage of the identified mutation based on the use of the clear-cell renal carcinoma 786-0 cells transfected with either empty (EV), WT VHL, or VHL-R167Q encoding vectors (Figure 1A) and showed that this mutation, despite the induction of *VHL* (Figure 1B), still induced HIF-2α expression in a marginal manner, as assessed by western blot, and that it was most likely associated with a decreased degradation of HIF-2 (Figure 1B). Such expression was confirmed using fluorescence microscopy that demonstrated staining of HIF-2α in this condition (Figure 1C,D).

We asked whether VHL R167Q interferes with cell morphology. As depicted in Figure 1D, compared with 786-0 EV used as control, cells transfected with WT-VHL or VHL-R167Q showed similar morphology. However, slight differences were noted between EV- and VHL WT/R167Q-transfected 786-O cells, with the latter exhibiting a more rounded and thinner morphology. The capacity of these cells to form colony was also examined. The 786-0 cells transfected with EV had a more pronounced potential to grow as colonies, compared with the WT VHL- and VHL-R167Q-transfected 786-O cells (Figure 1E,F). In addition, we calculated the average surface area covered by the cells spreading on the plastic substrate. We found that both WT VHL- and VHL-R167Q-transfected cells spread significantly less on the substrate than 786 EV cells, adopting a more fusiform/mesenchymal phenotype. (Figure 1G). These observations further highlight the tumor promoting function of VHL inactivation in these RCC models and also suggest that the expression of VHL-R167Q may be associated with specific features.

### 3.2. The R167Q VHL Mutation Interferes with Epithelial–Mesenchymal Plasticity

**Figure 2:**
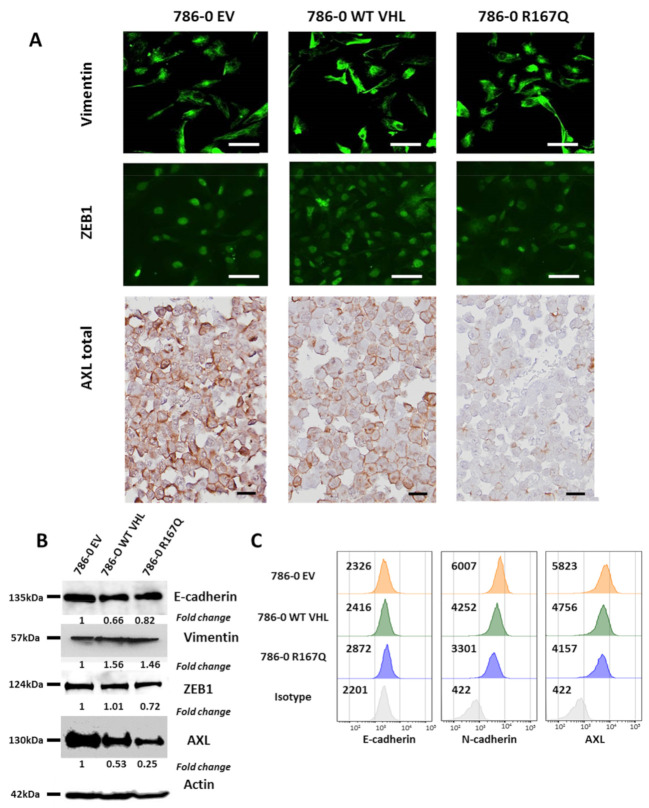
VHL gain of function in VHL-R167Q downregulates AXL expression. (**A**): Staining of vimentin, ZEB-1 and AXL by immunofluorescence (Scale bar, 100 μm) and immunohistochemistry (FFPE) (Scale bar 25 μm) on 786-0 EV, 786-0 WT VHL, and 786-0 VHL-R167Q. (**B**): Western blot analysis of E-cadherin, vimentin, ZEB-1, AXL and ACTIN in 786-0 EV, 786-0 WT VHL, and 786-0 VHL R167Q cells, the uncropped western blot figures in Figure S3. (**C**): Representative flow cytometry intensity histogram for E-cadherin, N-cadherin, and AXL on 786-0 EV, 786-0 WT VHL, and 786-0 R167Q cells. Isotype control (Ctrl IgG) is shown in gray.

As hypoxia has been reported to trigger epithelial-to-mesenchymal transition (EMT) in several types of cancer, we investigated the potential of the pseudo-hypoxic state induced by VHL-R167Q to modulate EMT-associated markers. We first focused on the expression of AXL, E-cadherin, vimentin and ZEB1. AXL is a well-known marker of mesenchymal differentiation in multiple types of cancer [24,25,26]. As depicted in Figure 2, using immunocytochemistry, western blot, and flow cytometry analysis, we found that 786-0 EV expressed high AXL levels, while both WT VHL- and R167Q-transfected 786-0 cells have a marked reduction in AXL expression, with R167Q-transfected 786-0 cells showing the lowest expression levels. These results further show the existence of an AXL expression gradient. This gradient may be linked with the VHL gradient and HIF-2α-dependent mechanisms. On the other hand, by immunofluorescence and western blot analysis, we found no differences (Figure 2A) or a slight increase (Figure 2B) in the expression of another well-known mesenchymal marker, vimentin, in (WT VHL and VHL-R167Q). Western blot analysis also revealed a slight decrease in ZEB1 expression in both WT VHL- and VHL-R167Q-transfected cells compared with EV-transfected cells (Figure 2B). Western blot analysis also showed no differences in global E-cadherin expression (Figure 2B). However, in flow cytometry experiments, a slight increase of cell surface E-cadherin was noted exclusively in VHL-R167Q-transfected cells. In this line, flow cytometry showed marked downregulation of N-cadherin expression in VHL-R167Q and WT VHL 786-O cells compared with 786-0 EV, with VHL-R167Q displaying the lowest levels (Figure 2C).

These observations suggest that fluctuations occur in the EMT state in these cells in a manner dependent on HIF2/VHL regulation. The expression of VHL-R167Q seems to be especially detrimental to the expression of certain mesenchymal markers, but not all, reinforcing the notion that the HIF2/VHL pathway is a strong mediator of epithelial–mesenchymal plasticity (EMP).

### 3.3. Comparison of Transcriptional Profile of WT VHL and VHL-R167Q Showed Discrete Differences

**Figure 3:**
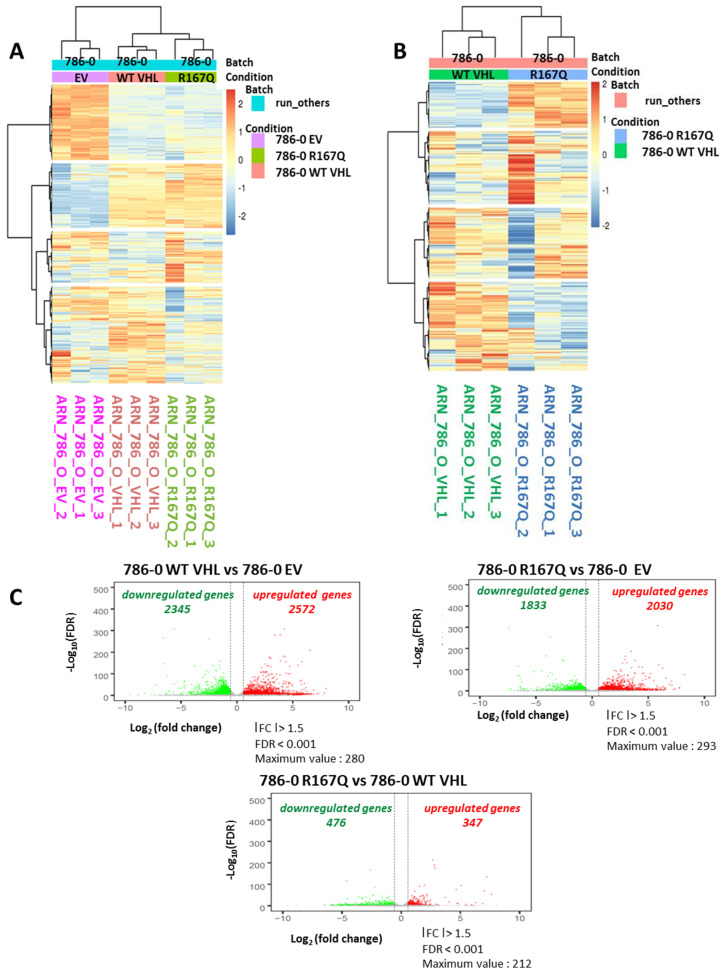
RNA-Seq analysis revealed discrete transcriptional differences between WT VHL and VHL-R167Q. (**A**): Heatmap from RNA-Seq analysis comparing 786-EV, 786-0 WT VHL, and 786-0 VHL-R167Q cells in triplicate. (**B**): Heatmap from RNA-Seq analysis comparing 786-0 WT VHL and 786-0 R167Q in triplicate. (**C**): Volcano plot of genes differentially expressed (Log_10_ fold change). Genes differentially expressed in 786-0 WT VHL vs. 786-0 EV, in 786-0 R167Q vs. 786-0 EV, and in 786-0 R167Q vs. 786-0 WT VHL are listed in Supplementary Materials Table S1. For 786-0 WT VHL vs. 786-0 EV (left), a total of 2345 downregulated genes and 2572 upregulated genes, with a fold change >1.5, FDR < 0.001 were identified. For 786-0 R167Q vs. 786-0 EV (right), a total of 1833 downregulated genes and 2030 upregulated genes were identified, with a fold change >1.5, FDR < 0.001. For 786-0 R167Q vs. 786-0 WT VHL (bottom), a total of 476 downregulated genes and 347 upregulated genes were identified, with a fold change >1.5, FDR < 0.001 and a maximum value at 212.

We next compared the transcriptomic profiles of transfected 786-0 cells—EV, WT VHL, and VHL-R167Q—using RNA-Seq. Triplicate samples were analyzed for each cell line. Data shown in Figure 3A,B indicate distinct transcriptomic profiles between EV-, WT VHL-, and VHL-R167Q-transfected cells (Figure 3A), as well between WT VHL- and VHL-R167Q-transfected cells (Figure 3B). Results depicted in Figure 3C illustrate a com-parative analysis performed to examine the transcriptomic profiles of WT VHL and VHL-R167Q cells and indicate a difference in terms of gene expression in these two cell lines. Volcano-plot analysis comparing WT VHL and EV indicated 2345 genes were downregulated, and 2572 genes were upregulated in WT VHL. Analysis of VHL-R167Q vs. EV identified 1833 genes that were downregulated and 2030 genes that were upregu-lated in VHL-R167Q. Finally, we compared the gene expression profiles of VHL-R167Q and WT VHL. Only 476 genes were found upregulated in VHL-R167Q com-pared with WT VHL, and 347 genes were found downregulated. Together, these data in-dicate im-portant differences between VHL null (EV) cells and other transfectants, whereas 786-0 cells transfected with WT VHL or VHL-R167Q exhibited discrete variations in their gene expression profile.

### 3.4. VHL-R167Q Mutation Modulates the Expression of Genes Belonging to Hypoxia and Stemness Pathways

**Figure 4:**
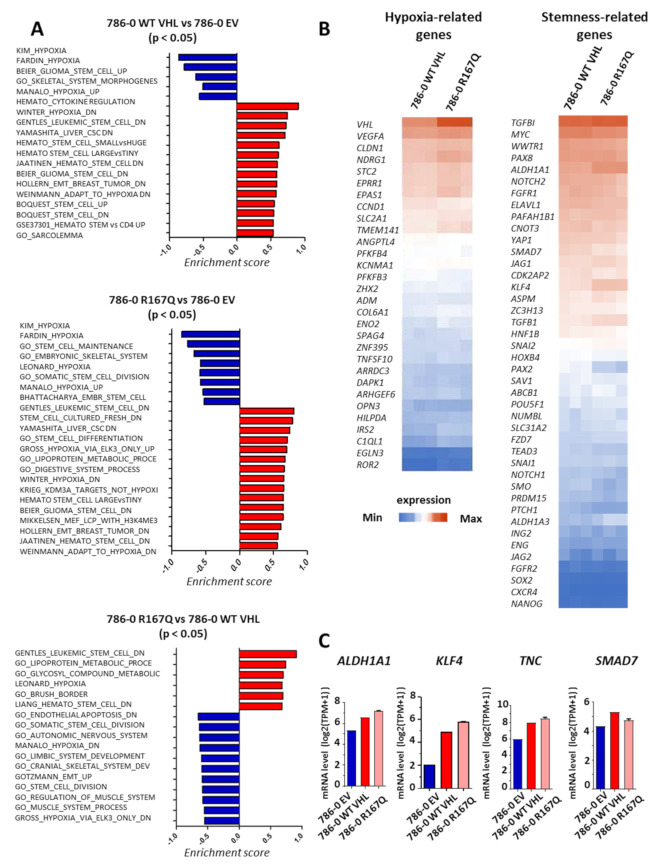
Hypoxia and stemness pathways are enriched in 786-0 VHL-R167Q, reflecting an increase in cell plasticity. (**A**): Differentially enriched gene sets nominated from GSEA analysis in 786-0 WT VHL vs. 786-0 EV, 786-0 R167Q vs. 786-0 WT VHL, 786-0 R167Q vs. 786-0 EV classified by enrichment scores. A positive score indicates enrichment, whereas a negative score indicates enrichment in the indicated conditions. (**B**): Heatmap displaying log2-transformed expression levels of a panel of hypoxia-related genes and stemness-related genes in 786-0 WT VHL and 786-0 R167Q. Heatmaps comparing the three models are presented in Figure S1. (**C**): mRNA expression of *ALDH1A1*, *KLF4*, *TNC*, and *SMAD7* from RNA-Seq analysis in the three 786-0 cell lines. The bar graphs show log2-transformed expressions.

In previous studies, we provided evidence indicating that hypoxia induces stemness features in cancer cells [27]. Here, we asked whether VHL-R167Q mutation, assumed to induce a pseudo-hypoxic state, also induced transcriptional changes associated with hypoxia. GSEA analysis integrating hypoxia-, EMT-, and stem cell-related signatures showed significant differences (enrichment scores with *p* < 0.05) between 786-0 WT VHL and 786-0 EV and between 786-0 R167Q and 786-0 EV, as well as between 786-0 R167Q and 786-0 WT VHL (Figure 4A). As expected, the R167Q mutation appeared to maintain a hypoxic state, as shown by enrichment of hypoxia signatures and related metabolic pathways. We noted the alteration of certain stem cell-related signatures but no significant alteration of EMT signatures. From this GSEA analysis, we extracted genes belonging to the enriched hypoxia- and stemness-related gene signatures and generated comparative heatmaps depicting the gene expression levels found in 786-0 WT VHL- and 786-0 R167Q-transfected cells (Figure 4B). Thus, we further investigated whether the VHL-R167Q mutation, through its capacity to induce pseudo-hypoxia, may result in the induction of genes associated with cancer cell stemness. We could distinguish genes that were upregulated in hypoxia and stemness pathways in 786-0 R167Q when compared with the 786-0 WT VHL condition. Namely, the expression of *ALDH1A1*, a well-known stemness marker [28,29], was gradually increased from 786-0 EV-, to WT- and 786-0 R167Q-transfected cells (Figure 4B,C). The same trend was observed for *KLF4* and *TNC* genes, encoding Kruppel-like factor 4 and tenascin C, respectively. Previous studies have implicated tenascin C in the regulation of stem cell maintenance and the promotion of the metastatic niche [30,31]. *KLF4* has been implicated in the regulation of development, cell plasticity, and cross talks with key signaling pathways, including TGF-β, Notch, and Wnt signaling [32]. *KLF4* was significantly upregulated in 786-0 R167Q compared with WT VHL-transfected cells, and its expression was minimal in the control 786-0 EV-transfected cells (Figure 4C). Interestingly, *SMAD7* expression, an attenuator the TGFB/SMAD2/3 pathway, was reduced in 786-0 R167Q- vs. WT VHL-transfected cells.

### 3.5. R167Q VHL Mutation Regulates Genes Associated with Poor Prognostic and Overall Survival

**Figure 5:**
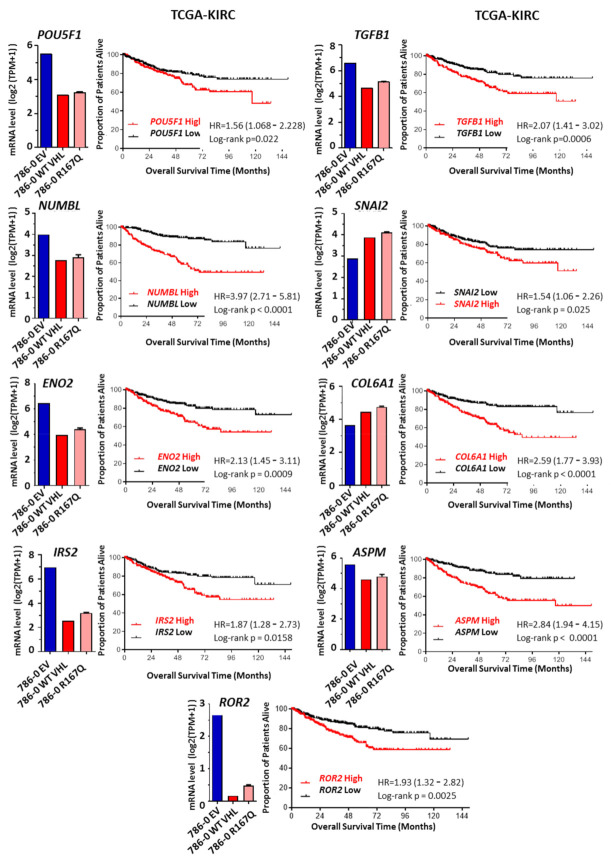
A set of VHL-regulated genes enriched in VHL-R167Q is associated with poor survival in the RCC TCGA dataset. Kaplan–Meier curves were generated to analyze the association between patient survival and mRNA expression of *ENO2*, *IRS2*, *ROR2*, *POU5F1*, *NUMBL*, *TGFB1*, *SNAI2*, *COL6A1*, *ASPM* genes in RCC tumors of the TCGA KIRC. Patients were stratified into two groups (low and high expression). The bar graphs show log2-transformed expression in the three 786-0 derivatives.

To test whether our findings in 786-0 cells are relevant in the clinical setting of renal clear cell carcinoma, we investigated RNA-Seq data from Kidney Renal Clear Cell Carcinoma (KIRC) of The Cancer Genome Atlas (TCGA). We systematically evaluated whether expression of any individual genes present in heatmaps (Figure 4) had a significant effect on disease-specific survival in RCC patients. Patients were stratified into two groups, with high or low gene expression based on median expression values. Kaplan–Meier plots were generated to assess the overall survival of patients, and curves were compared using the Mantel–Cox log-rank test. Among the hypoxia-regulated genes, *ENO2*, *IRS2*, and *ROR2* were associated with significantly worse survival (Figure 5). *ENO2*, also known as neuron-specific enolase, can be expressed by neuronal and cancer cells and may have an important role in glucose metabolism under hypoxic conditions [33,34]. *IRS2* (insulin receptor substrate-2) can be increased in cancer, including lung cancer, hepatocellular carcinoma (HCC), and breast and renal cancers, with the potential to regulate EMT pathways [35,36]. *ROR2* has been involved in the Wnt pathway in various cancer systems and in hypoxia-induced plasticity in melanoma cells [37,38,39]. Among the stemness-related genes, *POU5F1* (*OCT-4*), *NUMBL*, *TGFB1*, *SNAI2*, *COL6A1*, and *ASPM* were found to have significant effects on survival (Figure 5). *POU5F1* (*OCT4*) is known to be regulated by HIF2, and it is associated with poor prognosis in ccRCCs [40,41,42,43]. Unexpectedly, *KLF4* and *ALDH1A1* were positively associated with survival, whereas *TNC* and *SMAD7* showed no statistical differences (not shown). *NUMBL* is a close homologue of *Numb*, a Notch-associated regulatory factor with potential functions in regulating epithelial–mesenchymal plasticity [44,45]. *TGFB1* is known to have a central role in the regulation of EMT, fibrosis, stem cell maintenance, invasiveness and metastatic development [46,47,48]. *SNAI2*, also known as Slug, is a well-known EMT transcription factor that is critically involved in oncogenesis and in the maintenance of stemness and progenitor cells [49,50,51]. *COL6A1* is a member of the collagen family that is involved in numerous biological processes, including apoptosis, autophagy, stemness, differentiation, and tumor progression, including in RCC [52,53]. *ASPM* (abnormal spindle-like microcephaly-associated) has been implicated in a cell-fate determination of neuronal progenitor cells and in the development of malignant gliomas [54,55].

In the 786-0 cell derivatives, the expression of *ENO2*, *IRS2*, *ROR2*, *POU5F1*, *NUMBL*, *TGFB1*, and *ASPM* was generally higher in 786-0-EV compared with VHL-WT-transfectants and showed intermediate levels in VHL R167Q-tranfected 786-0 cells (Figure 5). These findings suggest that there is a strong VHL-dependent regulation of these genes, coinciding with the pseudo-hypoxic state of 786-0 transfectants, and that among VHL-regulated genes, those pseudo-hypoxic states may contribute significantly to the aggressiveness of RCC tumors. By contrast, *SNAI2* and *COL6A1* had increased expression in VHL-WT- and VHL-R167Q- compared with EV-transfected cells, and their expression was slightly increased in the VHL-R167Q transfectants as compared with their VHL-WT counterparts. In fact, this corroborates results obtained for *ALDH1A*, *KLF4*, and *TNC*, which suggests a more complex regulatory network and the presence of particular plasticity or stemness features acquired by VHL R167Q mutants, with potential impact on the survival of patients.

## 4. Discussion

Clear cell renal cell carcinomas are characterized by their hypervascularity and resistance to conventional anticancer treatments. An emerging role of VHL biology is its effects on the tumor microenvironment, and there is evidence that the VHL pathway targets the hypoxia-inducible factors (HIFs) family of transcription factors.

VHL inactivation leads to the constitutive stabilization of HIFs and to the increased expression of target genes involved in the unfavorable tumor microenvironment. Although studies by Ding et al. revealed how the R167Q VHL mutation contributes to tumorigenesis and identified a potential targeted therapy for ccRCC and other VHL-related disease in patients carrying R167Q mutation or similar missense mutations [17], the specific role of the R167Q VHL mutation and selective transcriptional regulation remains largely unknown. While some studies have shown that VHL-R167Q is deficient or partially deficient in downregulating HIF-2α [56], other studies have shown that it efficiently downregulates HIF-2α [57]. Here, using a VHL-mutated RCC cell line that express the R167Q VHL mutation in a doxycycline-inducible system, we demonstrated that this mutation is efficient in inducing a pseudo-hypoxic state. Our results are consistent with the notion that the R167Q mutation “only partly” mimics VHL deficiency, while having many of the salient features observed following WT VHL transfection.

VHL alterations are considered to be an early event in renal tumorigenesis and progression [58]. Moreover, it has been reported that mutations in the VHL gene refine histologic diagnostic criteria in RCC, serving as adjuncts to the present morphology-based diagnosis of RCC [59]. In the course of these studies, we asked whether R167Q VHL mutation influences cell line behavior. To this end, we examined morphology and colony formation and performed a comparative morphological analysis between WT VHL- and VHL-R167Q-reconstituted cells. We demonstrated that both cells display similar morphology, with decreased spreading, although a slight shift toward a more epithelial phenotype was observed, as illustrated by the downregulation of AXL and ZEB1 expression. Colony formation was similar in both cell lines. Our data suggest that aside from similar morphological and biological characteristics associated with this type of VHL mutation, VHL-R167Q-transfected cells showed a marginal HIF-2 expression, most likely associated with a decreased degradation. While it is well admitted that VHL mutations play a significant role in regulating the development, invasiveness, and survival characteristics of RCC, the role of R167Q VHL mutation in modulating tumor plasticity remains unknown. Therefore, we attempted to evaluate the impact of this mutation on EMP. We obtained data indicating that the R167Q mutation interferes with the EMP-associated phenotype in an HIF-2-dependent manner.

To obtain insight into the mechanism by which this mutation interferes with gene transcription, we performed a global transcriptional analysis and demonstrated that transfection by WT VHL and R167Q VHL mutation induced transcriptional changes in VHL R167Q. Interestingly, we showed the existence of a strong relation between genes regulated by hypoxia and those regulated by the R167Q VHL mutation, suggesting that the induced pseudo-hypoxia is effective in activation of hypoxia-associated events.

More importantly, our work provides another important novel aspect on the potential of this mutation on RCC plasticity, as we noticed that the R167Q VHL mutation regulates the expression of several genes belonging to distinct pathways involved in tumor plasticity, including stemness. We consider that the results obtained for 786-0 VHL R167Q-transfected cells do not demonstrate a strong link between this mutated form of VHL and a plain EMT. In fact, because the EMT process belongs to the large functional spectrum of cell plasticity, it is likely that EMT-, hypoxia-, and stemness-associated pathways are strongly overlapping, but their functional impact defines the relevance of more specialized and better defined pathways such as stemness and hypoxia response. Expression levels of several genes, including *SMO*, *SNAI2*, *TGFB*, *CNOT3*, *PAX2*, and *ALDH1A1*, are also linked to EMT pathways and were found to be increased in 786-0 R167Q. However, rather than a functional EMT, our observations support a link to stemness and hypoxia pathways reflecting an intermediate phenotype.

Aside from the well-known OCT-4 and TGF-β, evidence of differential expression in genes known to be regulated by TGF-β signaling, such as *TNC*, *KLF4*, *SMAD7,* or *SNAI2*, is intriguing. The evidence supports the notion that the R167Q VHL mutation is associated with a higher intrinsic TGF-β activity. In the TCGA samples, however, the expression of these genes was not systematically associated with poorer survival (e.g., *TNC*, *KLF4*, *SMAD7*). Further work is needed to clarify this finding. Importantly, our study provides supporting evidence that certain hypoxia-related genes are associated with a lower survival in RCC patients—that is, *IRS2*, *ROR2*, and *ENO2* [35,38,39]. Moreover, the RNA-Seq analysis coupled with the interrogation of the TCGA KIRC dataset allowed the identification of novel candidates strongly associated with poor prognosis and the lethal evolution of RCC, including *NUMBL*, *COL6A1*, and *ASPM*, as well as *SNAI2* [51]. The specific impact of each gene in the setting of RCC, hypoxic, and pseudo-hypoxic contexts should be investigated in future studies. Further work is warranted to assess the links between the expression of these genes and patient survival, as well as patient response to treatments in the setting of early and advanced diseases. One unresolved question concerns the relationship between the expression of these genes and the type of VHL alterations found in the tumor samples. Even if the VHL mutation R167Q is considered a common type 2B missense VHL mutation in hereditary VHL disease, it remains a rare event in sporadic RCC. Thus, it would be interesting to investigate the potential effects of these genes based on VHL status (non-inactivated vs. inactivated) or considering the different VHL mutation subtypes.

## 5. Conclusions

In conclusion, several studies have demonstrated that the hypoxic microenvironment drives the selection of a more aggressive cancer cell population through cellular adaptations referred to as epithelial–mesenchymal plasticity. On the basis of global gene expression profiling, our data demonstrate that the R167Q VHL mutation leads to molecular changes indicative of more pronounced stem cell traits and that the mutation is a presumed source of tumor heterogeneity and resistance. These data would suggest that the R167Q VHL mutation induces a precisely tuned dysregulation of genes associated with a tumor phenotype towards increased RCC plasticity. Findings are in support of the notion that such a mutation may provide further RCC advantage, and more interestingly, may influence tumor survival. This research should guide the development of innovative targeted therapy to treat RCC by stabilizing R167Q and other related mutations.

## Data Availability

RNA Seq data presented in this study are available on request from the corresponding author.

## Supplementary Materials

The following are available online at https://www.mdpi.com/article/10.3390/cancers13153897/s1, Figure S1: Heatmap displaying log2-transformed expression levels of a panel of hypoxia-related genes and stemness-related genes in 786-0 EV, 786-0 R167Q, and 786-0 WT VHL, Figure S2: The uncropped western blot figures of Figure 1B, Figure S3: The uncropped western blot figures of Figure 2B, Table S1: List of genes differentially expressed in 786-0 EV vs. 786-0 R167Q, in 786-0 EV vs. 786-0 WT VHL, and in 786-0 WT VHL vs. 786-0 R167Q.
