# Supplementary material for: The Most Common VHL Point Mutation R167Q in Hereditary VHL Disease Interferes with Cell Plasticity Regulation
Source: Cancers (Basel). 2021 Aug 2;13(15):3897. doi: 10.3390/cancers13153897 (PMC8345752; doi:10.3390/cancers13153897)
Supplement: Supplementary file 1 [file cancers-13-03897-s001.zip › cancers-1304810-supp-final.pdf]

Supplementary Materials: The Most Common *VHL* Point Mutation R167Q in Hereditary VHL Disease Interferes with Cell Plasticity Regulation

Stéphanie Buart, Stéphane Terry, M'boyba Khadija Diop, Philippe Dessen, Sophie Couvé, Abdérémane Abdou, Julien Adam, Jérôme Thiery, Pierre Savagner and Salem Chouaib

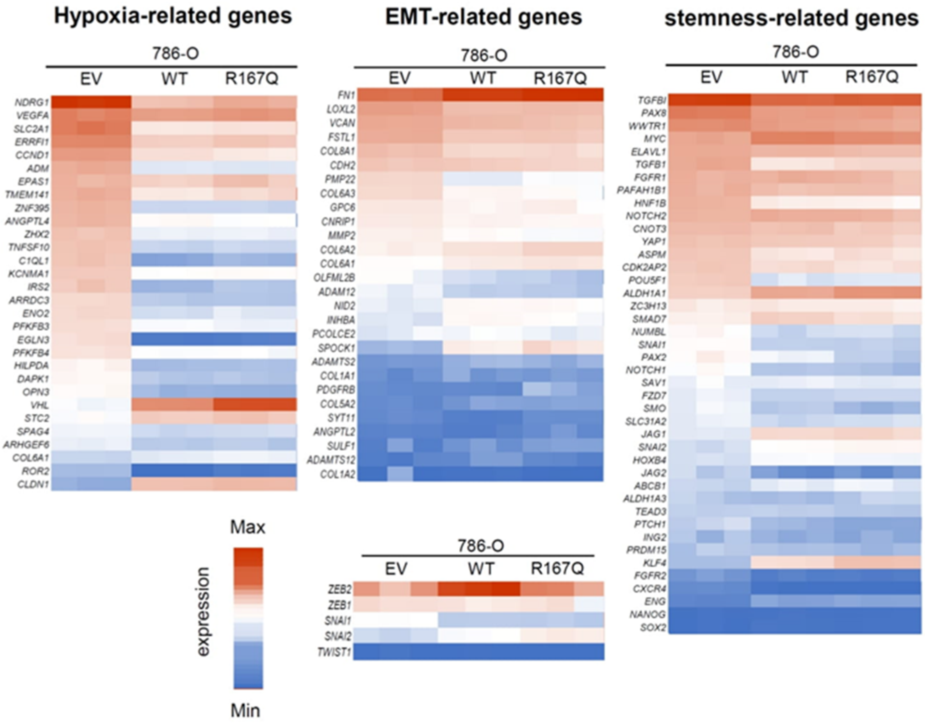

Figure S1. Heatmap displaying log2-transformed expression levels of a panel of hypoxia-related genes and stemness-related genes in 786-0 EV, 786-0 R167Q 786-0 WT VHL and 786-0 R167Q.

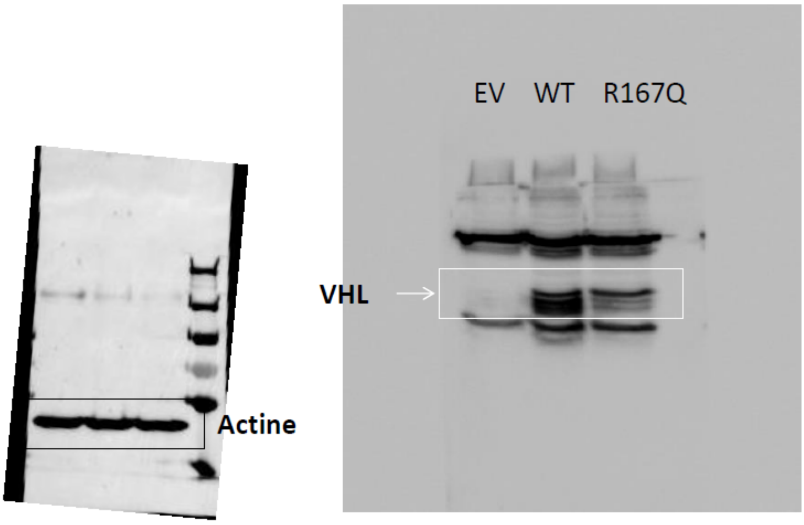

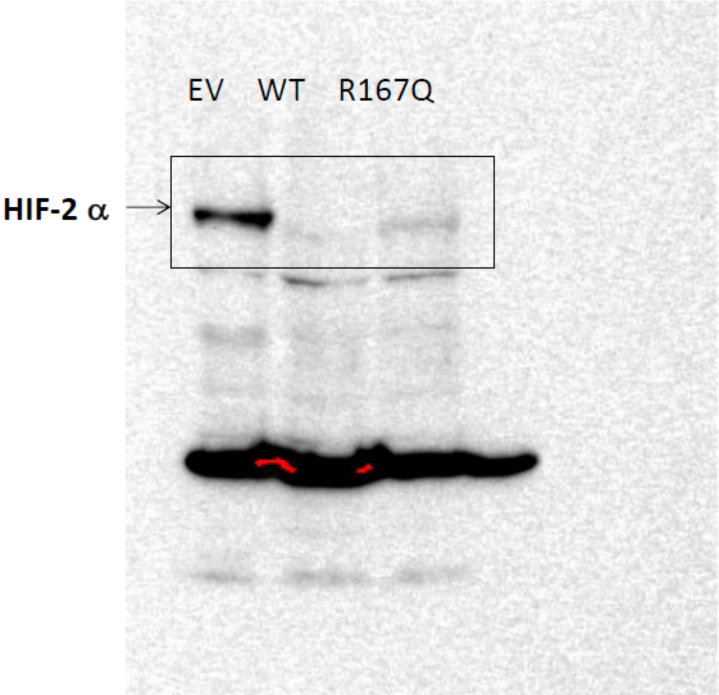

Figure S2. The uncropped western blot figures of Figure 1B.

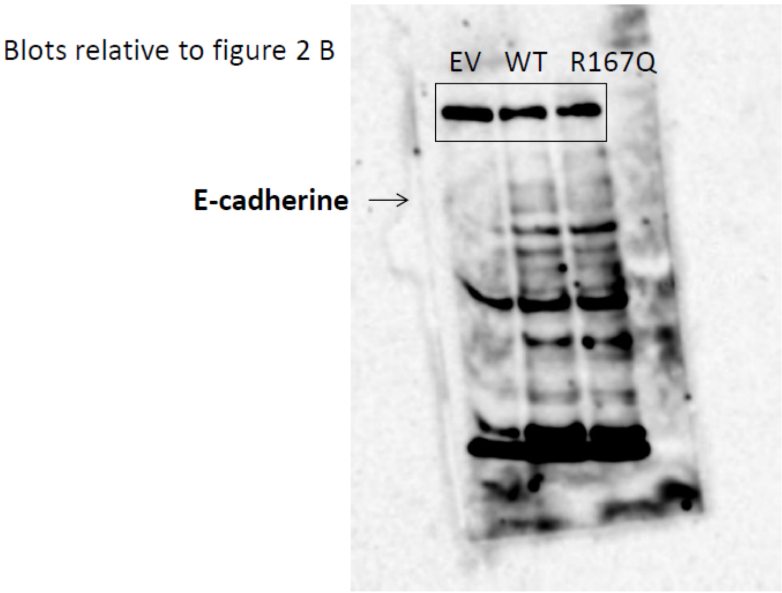

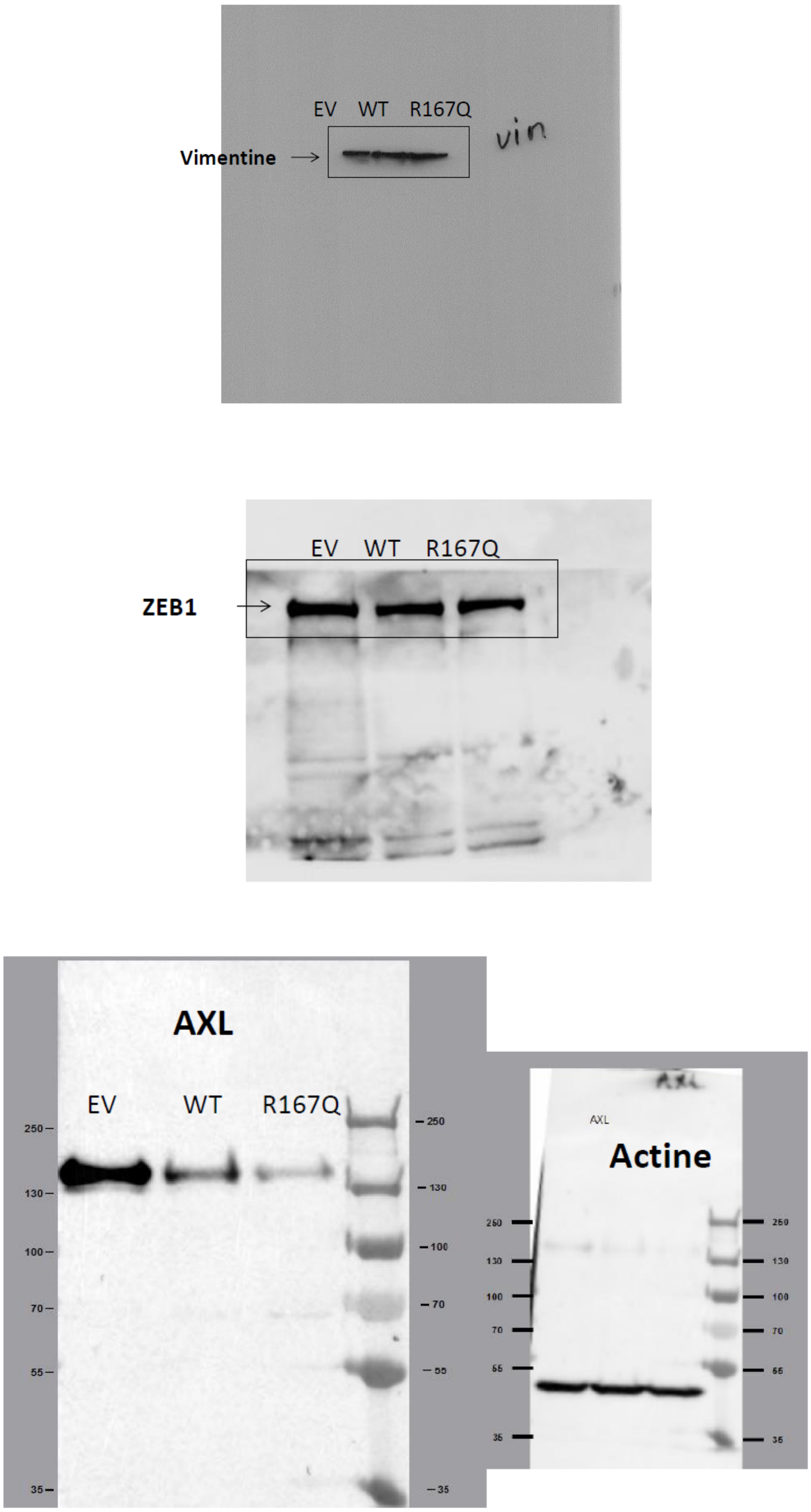

Figure S3. The uncropped western blot figures of Figure 2B.
